# An Analytical Multiple-Temperature Model for Flash Laser Irradiation on Single-Layer Graphene

**DOI:** 10.3390/nano10071319

**Published:** 2020-07-05

**Authors:** Anca M. Bucă, Mihai Oane, Ion N. Mihăilescu, Muhammad Arif Mahmood, Bogdan A. Sava, Carmen Ristoscu

**Affiliations:** 1 Faculty of Physics, University of Bucharest, 077125 Măgurele, Ilfov, Romania; anca.buca@yahoo.com (A.M.B.); arif.mahmood@inflpr.ro (M.A.M.); 2 National Institute for Laser, Plasma and Radiation Physics, 077125 Măgurele, Ilfov, Romania; mihai.oane@inflpr.ro (M.O.); ion.mihailescu@inflpr.ro (I.N.M.); bogdan.sava@inflpr.ro (B.A.S.)

**Keywords:** Multiple-Temperature Model, Zhukovsky’s approach, graphene, comparison with other theoretical models

## Abstract

A Multiple-Temperature Model is proposed to describe the flash laser irradiation of a single layer of graphene. Zhukovsky’s mathematical approach is applied to solve the Fourier heat equations based upon quantum concepts, including heat operators. Easy solutions were inferred with respect to classical mathematics. Thus, simple equations were set for the electrons and phonon temperatures in the case of flash laser treatment of a single layer of graphene. Our method avoids the difficulties and extensive time-consuming nonequilibrium green function method or quantum field theories when applied in a condensed matter. Simple expressions were deduced that could prove useful for researchers.

## 1. Introduction

Graphene is a two-dimensional (2D) material, consisting of a hexagonally arranged lattice of carbon atoms. The term “graphene” is fully functional for a solitary deposit of graphite. Most of the thermal properties of Graphene are derivatives of graphite, and allow the inscription of the exceedingly anisotropic nature of this material [1]. For applications, graphene transistors and interconnects benefit from the high in-plane thermal conductivity, up to a specific channel length. Nonetheless, because of its weak thermal coupling to the substrate, dissipation through interfaces and contacts produces a bottleneck. Heat flow in graphene or graphene composites could likewise be tunable through a variety of means, including phonon dispersing by substrates, edges, or interfaces. The thermal properties of graphene originate from its 2D nature, shaping a rich area for new disclosures of heat flow physics and conceivably prompting novel thermal management applications [2].

Electron–Phonon (E–P) coupling is the main phenomenon that intermediates Joule heating, laser–material interactions, and hot electron relaxation. A complete understanding of E–P coupling phenomena is critical to understanding the material properties and improving electronic devices [3,4,5,6,7,8,9,10]. Boltzmann transport equation [11,12,13] and a combination of molecular dynamics with modal analyses [14,15] are accurate methods, but they are complicated, which hinders their use by researchers. A simple Multiple-Temperature Model (MTM) has been developed, which is an extension of the Two-Temperature Model (TTM) [7,16]. For phonons that combine powerfully, a more demanding way is to unswervingly treat the scattering. The cost of the simulation is high; however, MTM can still be used by allowing the phonons to interact with a common thermal reservoir under relaxation time approximation. The MTM models in the literature are either in complex forms that hinder the easy implementation or only available in the steady-state form.

In this study, the MTM for flash laser experiments in the case of a Single Layer of Graphene (SLG) is combined with Zhukovsky’s approach to heat equations. Simple expressions for the electron and phonon temperatures during flash laser experiments on single-layer graphene are developed.

## 2. Basic Statement 

The Two-Temperature Model (TTM) was developed by Anisimov and collaborators [17]. From a theoretical point of view, a few attempts have been presented to improve or simplify the TTM model [18,19]. Very recently [20], a more complex model was pointed out that replaced the standard TTM. The new model, named “Multiple-Temperature Model (MTM)”, is more elaborated from theoretical point of view and provides a much better fitting of experimental data, e.g., in predicting the thermal field during laser-graphene interaction. For single-layer graphene, (a) longitudinal optical phonons (LO), (b) transverse optical phonons (TO), (c) out-of-plane optical phonons (ZO), (d) longitudinal acoustic phonons (LA), (e) transverse acoustic phonons (TA), and (f) out-of-plane acoustic phonons (ZA) have been identified. TTM uses the Quantum-Espresso package or/and EPW (Electron–Phonon coupling using Wannier functions) for simulations based on first principles density functional perturbation theory (DFPT) and an MTM [7]. The main goal of the current study is to develop a less sophisticated MTM using the Zhukovsky’s formalism [20,21,22], thus producing innovation in current field. 

The classical TTM formalism can be expressed as

(1) $$A.T_{e}\left(\frac{dT_{e}}{dt}\right)=K\left(\frac{\partial^{2}T_{e}}{\partial x^{2}}+\frac{\partial^{2}T_{e}}{\partial y^{2}}+\frac{\partial^{2}T_{e}}{\partial z^{2}}\right)-G\left(T_{e}-T_{p}\right)+P_{a}\left(\vec{r},t\right)$$

and

(2) $$C_{e}\left(\frac{dT_{p}}{dt}\right)=G\left(T_{e}-T_{p}\right)$$

Here, *T_e_* and *T_p_* are the electrons and phonons temperature; *t* is time; *x*, *y* and *z* are spatial coordinates; and $\vec{r}$ is the radius/distance vector. *G* is the coupling factor between electrons and phonons. *P_a_* is the heat source that is induced through laser–SLG interaction. The interaction could be of classical or steady-state quantum mechanical type. *A* is the electron heat capacity, *K* is the electrons thermal conductivity of the graphene, and *C_p_* is electron heat capacity of the graphene. MTM in our formalism has the following analytical form (for the phonon branch *i*, and E–P coupling with coupling factor *G_ep,i_*):

(3) $$A.T_{e}\left(\frac{dT_{e}}{dt}\right)=K_{e}\left(\frac{\partial^{2}T_{e}}{\partial x^{2}}+\frac{\partial^{2}T_{e}}{\partial y^{2}}+\frac{\partial^{2}T_{e}}{\partial z^{2}}\right)-\sum G_{ep,i}\left(T_{e}-T_{p,i}\right)+P_{a}\left(\vec{r},t\right)$$

and

(4) $$C_{e}\left(\frac{dT_{p,i}}{dt}\right)=K_{p,i}\left(\frac{\partial^{2}T_{p,i}}{\partial x^{2}}+\frac{\partial^{2}T_{p,i}}{\partial y^{2}}+\frac{\partial^{2}T_{p,i}}{\partial z^{2}}\right)+G_{ep,i}\left(T_{e}-T_{pi}\right)+G_{pp,i}\left(T_{lat}-T_{p,i}\right)$$

The links between the two models are the following equations:

(5) $$G=G_{e,p}=\sum G_{ep,i}$$

and

(6) $$\sum G_{pp,i}\left(T_{lat}-T_{p,i}\right)=0$$

where *G_pp_*_,*i*_ is the *p-p* coupling factor between phonon branch *i* and the scattering lattice reservoir, and *T_lat_* is the lattice reservoir’s temperature, defined as to ensure the energy transfer among phonon branches conservation. Until now, we have presented the general formalism of thermal effects in laser-graphene interaction. Our main goal was to solve analytically the general formalism described above in order to predict the thermal field in the laser–graphene interaction, as accurately as possible.

## 3. Linking the Zhukovsky’s Formalism and MTM: A Novel Approach

To the best of our knowledge, there are several articles available on “Zhukovsky’s formalism and MTM” approach [20,21,22], but no attempt has been carried out to link the two approaches with integral transform technique. The results are similar to the other studies but computational technique is unique, simple, and less time-consuming. It recognizes the novelty of this study. According to Ref. [20], linear Fourier equation can be written as

(7) $$\frac{\partial T}{\partial t}=a\frac{\partial^{2}T}{\partial x^{2}}+2b\frac{\partial T}{\partial x}+cx\;T\left(x,t\right)+dT\;\;\;$$

We set

(8) b=c=0

and

(9) a=γ

and

(10) $$d=-\frac{G_{ep}\gamma }{K_{e}}$$

In Equations (9) and (10), γ is the graphene thermal diffusivity.

In case of flash laser experiments, one may write for the heat source

(11) $$T\left(x,0\right)\approx S_{laser}\cdot \delta \left(t\right)$$

where

(12) $$S_{laser}=S_{0}\cdot exp\left(-S_{1}\cdot x^{2}\right)$$

with

(13) $$min\;\left\{\;S_{0},\;S_{1}\right\}>0$$

At the beginning of irradiation in flash laser experiments, we have the following approximation

(14) $$\;T_{e}\gg T_{P}$$

Using Equation (5), we may express the electron temperature as

(15) $$T_{e}\left(x,t\right)=e^{-\frac{G_{ep}\gamma }{K_{e}}\;\cdot \;t}\cdot \frac{S_{laser}}{2\sqrt{\pi \gamma t}}\;\int_{-\infty }^{+\infty }e^{-\frac{{\left(x-\xi \right)}^{2}}{4t\gamma }}\cdot \delta \left(\xi \right)d\xi \;$$

For phonons, we consider the following heat equation, where the source term is given by a mean electrons temperature:

(16) $$C_{p,i}\frac{\partial T_{p,i}}{\partial t}=\frac{\partial^{2}T_{p,i}}{\partial x^{2}}-G_{ep,i}\cdot T_{p,i}+G_{ep,i}\cdot {\bar{T}}_{e}$$

A “parallel” is then made between the heat equation in standard form and the one in Zhukovsky form. For this, we set

(17) b=c=0

and

(18) $$a=\frac{1}{C_{p,i}}$$

and

(19) $$d=-\frac{G_{ep,i\;\;}}{C_{p,i}}$$

Here, *i* denotes different kinds of phonon branches:

(20) i={ LA, TA, ZA, LO, TO,ZO }

The final result [20] for the phonons temperature of branch *i* ($C_{p,i}$ is the heat capacity of the phonons of branch *i*) is

(21) $$T_{p,i}\left(x,t\right)=e^{-\frac{G_{ep,i}\;}{C_{p,i}}\cdot t}\cdot \frac{G_{ep,i}}{C_{p,i}}\;\cdot {\bar{T}}_{e}\cdot \overset{+\infty }{\underset{-\infty }{\int }}e^{-\frac{{\left(x-\xi \right)}^{2}}{4t}\cdot \;C_{p,i}}\;\cdot d\xi \;$$

## 4. Simulations

**Figure 1:**
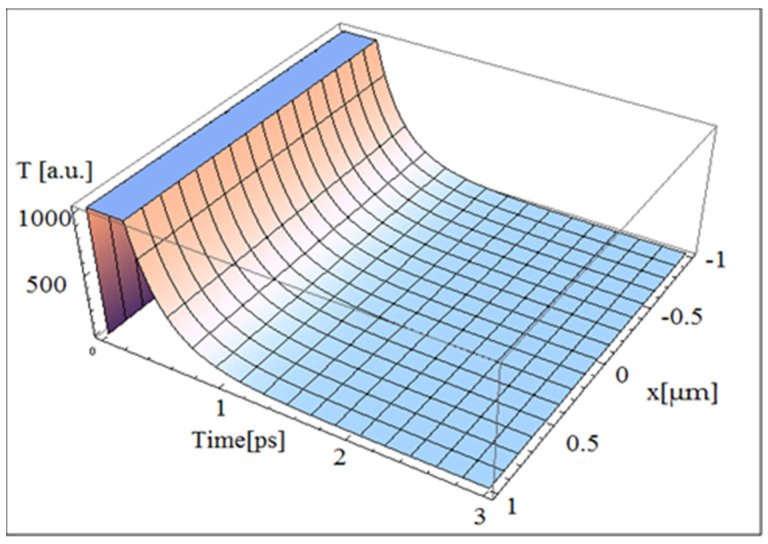
The electrons temperature versus current coordinate and time during flash laser irradiation of a single-layer graphene.

The input parameters given in Refs. [7,20] have been used for simulations: heat capacity *C*_e_ of 3.6 × 10^2^ J/m^3^K and thermal conductivity *K_e_* of 50 W/mK (see also Table 1 from Ref. [20]). The only exception is the laser intensity factor *S*_1_, which is equal to unity, in order to have arbitrary units. A laser spot radius equal to 0.5 µm and pulses of 25 fs have been taken into account. For the electron temperature, according to Equation (15), the 3D graphic, simulated via Mathematica software, is presented in Figure 1. One should notice that here, and in sequel, *T* stands for temperature variation rather than the absolute temperature. Figure 1 is in very good agreement with 2D graphics in Ref. [20]. In current simulation, the electrons are heated for a very short time (tenth of fs) to the maximum temperature, similar to data in Ref. [7], but the cooling time took, more realistically, about 0.5 ps, compared to about 0.15 ps in Ref. [7]. 

**Figure 2:**
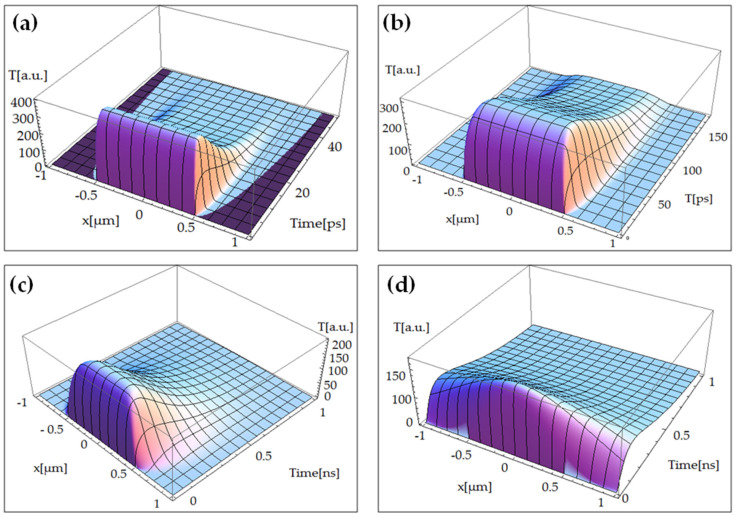
Temperatures field versus current coondinate and time during flash laser irradiation of a single-layer graphene of the (**a**) transverse optical phonons (TO), (**b**) longitudinal optical phonons (LO), (**c**) longitudinal acoustic phonons (LA), and (**d**) transverse acoustic phonons (TA) phonons.

Figure 2a shows the phonon’s temperature (using Equation (21)), based upon current model, for transverse optical (TO) phonons branch. It can be observed that where the laser beam intensity is zero, the temperature distribution is zero. This shows the perfect coupling between Zhukovsky’s formalism and Multiple-Temperature Model (MTM). However, thermal effects can be observed clearly beyond laser beam spot size (=1 µm), as a very short laser–graphene interaction time has been choosen (of ps order). The same phenomena can be analyzed in-case of Figure 2b,c realed to longitudinal optical phonons(LO) and longitudinal acoustic phonons (LA) braches, respectively. As the simulation time has been increased, from ps to ns, the heat-wave finds enough time to transfer from the irradiated spot to the rest of the target. This result has been presented in Figure 2d, in case of TA branch. These results prove the trustworthiness of our modeling approach. Figure 2a–d shows the coupling of laser beam in different branches (TO, LO, LA, and TA): higher the coupling factor, the greater the temperature field intensity. It divides the results into two groups, mathematically, as follows: (i) TO and LO, and (ii) LA and TA.

The output of current model is in good agreement with the one presented in Refs. [7,20]. The observation from Ref. [7] that electrons cool-down faster than all kinds of phonons (not only optical ones) is also confirmed by current simulation. It is due to similar reasons, related to *K_e_* and *C_e_* values, which are higher or similar (*K_e_*) to and much smaller (*C_e_*) than the phonon ones. The current simulation has also the advantage that it accurately indicates the thermal decrease of acoustic phonons, which is not very pronounced in Ref. [7]. The cooling time for acoustic phonons is higher, of the order of ns vs. hundred of ps. Accordingly, the present simulational analysis is more accurate than the one presented in Refs. [7,20]. It can be identified that the higher the coupling constant between electrons and phonons of kind *i*, the better the concordance between current model and the one presented in Refs. [7,20]. When the coupling constant is zero, the present model gives, as expected, a zero temperature variation, which is not the case in Refs. [7,20]. These results have been identified as classical results, while in Refs. [7,20], because of considering the Quantum-field effect, the temperature is not zero despite having the coupling-constant equal to zero. This is due to the Quantum-fluctuations of the vaccum and the laser beam. This study extended the original TTM into a MTM, by using phonon branch-resolved electron to phonon coupling factors. The results showed that different phonon branches are in strong nonequilibrium. Moreover, a comparison with TTM reveals that under steady state condition, MTM estimates 50% and 80% increase for electrons and phonons temperature, respectively [20]. 

## 5. Limitations of Current Model

The equations used in the present model have a “classical form”. Nevertheless, the solutions were figured out under quantum mechanical manner. For example, the heat operator is used to solve the heat equation.The following limitations were identified with respect to the application of this approach:

- The irradiation time/pulse duration should be superior to *1 fs*. If simulations are carried out for *as* pulse durations, the laser wavelength is quite close to the separation distance between nucleus and first electron and the concept of the temperature distribution becomes meaningless.
- The target size is confined to the (20–100) nm range. If the particle size is smaller than 20 nm, the Fourier law collapses and cannot provide reliable results. For more than 100 nm, the restriction to nano-object is infringed.
- In current study, single-layer absorption has been used, thus limiting our study to single layer graphene. For multilayer graphene, the analysis becomes intricate and difficult to calculate since the absorption law and heat-transfer coefficients, in real-time, should be reconsidered for each layer, demanding a quantum-field theory in solid-state treatment. This subject is laborious to handle in simulations.

## 6. Conclusions and Outlook

A new model for MTM applied to flash laser experiments on single-layer graphene is proposed. The results are found to be in good agreement with those reported in the Refs. [7,20]. The current model is, however, much simpler than previous ones, and is able to reproduce 3D thermal fields instead of only 2D ones. This model could be, therefore, an excellent and handy tool for researchers. The crux of the mystery of the current model is that we have applied a very powerful thermal model, namely, the Zhukovsky model [21,22,23], which has encoded in its parameters a lot of information. This work can be extended to the higher laser intensities. The classical heat transfer can be applied until the limit where nonlinear effects of interaction appear at 10^21^ W/cm^2^ [24]. Further, this model could be extended to other types of graphene such as graphene oxide or reduced graphene oxide. One should however introduce, in this case, the appropriate values of thermal properties of the target, resulting in modified temperature field distribution of the respective material. This technique is unique, simple, and less time-consuming (about 2 min using Core i7, 8th generation, and 16 GB Ram).
